# Supervision of Task-Shared Mental Health Care in Low-Resource Settings: A Commentary on Programmatic Experience

**DOI:** 10.9745/GHSP-D-18-00337

**Published:** 2019-06-24

**Authors:** Christopher G. Kemp, Inge Petersen, Arvin Bhana, Deepa Rao

**Affiliations:** a Department of Global Health, University of Washington, Seattle, WA, USA.; b School of Applied Human Sciences, University of KwaZulu-Natal, Durban, South Africa.; c South African Medical Research Council, Health Systems Research Unit, Durban, South Africa.; d Department of Psychiatry and Behavioral Sciences, University of Washington, Seattle, WA, USA.

## Abstract

Task-shared mental health care programs in low-resource settings often incorporate supervisory structures that would be difficult to implement at scale, and many rely on foreign specialist experts as supervisors. Future programs could leverage peer supervision, technology, competency assessments/fidelity checklists, and other tools. Mental health care specialists will require training, support, and incentives to supervise generalist care providers.

## INTRODUCTION

Mental disorders are the leading cause of years lived with disability globally.^1^ Yet in low- and middle-income countries (LMICs) and other low-resource settings, 75% of people in need of treatment for mental disorders never receive care.^2^^,^^3^ Effective services that are feasible, scalable, and sustainable in the context of critical shortages of financial and human resources are needed to bridge this treatment gap.^4^^–^^6^ Few health systems in LMICs can rely exclusively on specialists to deliver mental health interventions, nor can they afford to develop mental health programs in parallel to other services.^7^ Instead, they have to rely on existing cadres of health care workers and constrained financial resources to expand access for mental health services.

One promising approach has been to deliver psychosocial or pharmacological services via task sharing. Task sharing is an arrangement in which generalists—nonspecialist health professionals, lay workers, affected individuals, or informal caregivers—receive training and appropriate supervision by mental health specialists and screen for or diagnose mental disorders and treat or monitor people affected by them.^8^ Systematic reviews of task-shared mental health services in low-resource settings have demonstrated that the approach can be acceptable and feasible and can lead to substantial improvements in patient health outcomes, even in settings with few available specialists.^9^^,^^10^

To ensure generalist providers adopt evidence-based mental health services and deliver them with fidelity, task-sharing programs must incorporate effective systems for ongoing training, supervision, and mentorship.^11^ Initial trainings are a necessary but insufficient step toward building the confidence and competence of mental health clinicians.^12^ Supervision and mentorship are essential to developing the feedback loops that correct negative behaviors and reinforce positive behaviors as part of the cycle of learning, doing, and reflecting.^13^ In high-resource settings, supervision has been widely recognized as key to the development of a clinician's skills.^14^ Programs without ongoing supervision have been found to have low intervention fidelity and clinician competency,^15^ and established programs without supervisory support can risk significant declines in service delivery within 2 years.^16^ Indeed, the type of training received has been shown to be less important than the dosage of supervision in predicting clinician adoption of and fidelity to evidence-based mental health services.^17^ Supervised mental health clinicians receive essential emotional support and are less likely to experience burnout.^18^^,^^19^

The relative effectiveness of different supervisory models for task-shared mental health services in low-resource settings remains understudied, although recent calls for research suggest that a change is imminent.^20^^,^^21^ Little is known about the range of supervisory models already developed and implemented as part of task-shared mental health care in low-resource settings.^22^ An exploration of these models would offer support to future programs as staff plan, design, and implement task-shared programs. Our objectives were to provide an overview of the literature on the supervision of frontline and mental health care workers in low-resource settings, to describe and draw lessons from the experiences of implementers of task-shared mental health services in these settings, and to offer evaluative commentary for consideration by future investigators and implementers.

## OVERVIEW OF SUPERVISION MODELS

Supervision of frontline health care workers—including but not limited to those delivering mental health care—may take many forms. Most broadly, supervision refers to the cyclical process in which a senior professional or team sets expectations for the practice of health care workers at a lower level in the health system, observes and/or audits that practice, assesses whether it meets expectations, and provides guidance or takes corrective action.^23^ Supervisors employ a wide range of activities to carry out these functions, and health systems may focus on and prioritize some supervisory functions over others. Depending on that focus, models for supervision fall along a spectrum of 3 general categories: traditional supervision, supportive supervision, or mentorship.^24^

Supervision of frontline health care workers falls within 3 general categories: traditional, supportive, or mentorship.

Traditional supervision is distinguished by its focus on oversight for the purpose of identifying problems, with little emphasis on guidance or support. Many LMIC health systems can trace the use of this form of supervision to colonial powers that enforced rigid hierarchies with the goal of ensuring compliance among local staff and lower-level workers.^25^ Traditional supervision imposes the needs of the health system onto providers, while often failing to address the needs of providers or the needs of the patient population.^23^ The premise is that satisfactory performance can only be achieved through close control and punitive measures. Problem solving is reactive and episodic, rather than proactive and continuous.^26^ Under this model, frontline health care workers are not empowered to identify and solve issues on their own. Traditional supervision typically occurs through short visits by external supervisors to health care facilities and the completion of routine forms and checklists.^27^

In contrast, supportive supervision has been described as a process of strengthening relationships within the health system, with a focus on continuously identifying and resolving problems, optimizing the allocation of resources, and promoting teamwork and open communication, all with the goal of improving quality across all levels of the health system.^23^ Supportive supervision incorporates self-assessment and assessment by peers, as well as community input, shifting the locus of supervision from the supervisor-supervisee dyad to the entire workforce.^23^ It uses a practical system of objective measures to foster improvements in the procedures, personal interactions, and management in primary care facilities.^28^ Supportive supervision has been shown to be conducive to improvements in health worker performance and to a more general strengthening of health systems in low-resource settings.^23^ In sub-Saharan Africa, evidence suggests that supportive supervision increases job satisfaction and worker motivation.^29^ Supportive supervision is considered best practice and is the model specifically recommended in the context of task sharing; World Health Organization (WHO) guidelines state that supportive supervision should be regularly provided to all task-shared health workers, and that supervisors should themselves be competent and have appropriate supervisory skills.^30^

At the far end of the supervision spectrum, mentorship broadens the focus from building skills related to a specific intervention or health issue to fostering the development of a learner's professional career.^24^ Mentorship is holistic and longitudinal. It is a flexible teaching and learning process based on mutual trust and a shared set of learning objectives.^31^ Power is shared between mentors and mentees; mentees are free to direct their learning and identify and solve issues on their own. Evidence from LMICs suggests that mentorship contributes to improved quality of care outcomes.^24^ In high-income countries, mentorship has been found to be an effective intervention for knowledge translation.^32^ Cultural congruency between mentor and mentee may be important to ensuring an effective mentoring relationship.^33^^,^^34^

In addition to the general categories outlined above, supervision of frontline health care workers—including mental health care workers—can vary by dosage, mode or level, tools used, and the amount of supervisor training. Limited evidence of the comparative effectiveness of the various combinations of each of these variables exists; even the effectiveness of supervision compared to no supervision remains unclear and understudied.^35^^,^^36^ No ideal dosage of supervision has been identified—whether weekly, monthly, yearly in frequency, or 1 hour, 1 day, or 1 week in duration—although evidence does suggest that the quality of supervision is more important than its frequency.^37^ Peer supervision has been found to allow for collaboration and empathy outside the traditional supervisory hierarchy,^38^ and it may be less costly than other forms of supervision.^39^ Group-based supervision, facilitated by a supervisor, allows for increased coverage of supervision at reduced cost.^37^ Decision support tools and job aids can be used to structure supervision, and they have been shown to lead to faster supervisor reactions to shifts in supervisee behavior.^27^ Short checklists have also been linked to improvement in supervisee performance.^37^ Supervisors clearly need and benefit from training in the skills specific to supervision, although again no ideal amount or method of supervisor training has been identified.^37^

Supervision of frontline health care workers varies by dosage, mode or level, tools used, and supervisor training.

Specific to task-shared mental health care in low-resource settings, the literature offers few in-depth descriptions of models of supervision and even less evidence of comparative effectiveness. One early report, published in 2001, contains brief overviews of supervisory structures for pharmacological mental health care services integrated with primary care in Guinea-Bissau, India, Iran, and Nicaragua, and emphasizes the importance of ongoing supervision beyond the period of training.^40^ A series of case studies from Latin America and the Caribbean and sub-Saharan Africa, describing the implementation of both psychosocial and pharmacological task-shared services, specifically highlights the role of supervision in helping generalists who report feeling depressed or stressed when they start delivering mental health care interventions. These case studies also suggest that a minimum number of mental health specialists are required to supervise generalists and to provide specialized referral treatment services.^41^^,^^42^ Systematic reviews and cross-country studies of task-shared psychosocial and pharmacological services reiterate the need for ongoing supervision at the community and primary care levels to help generalists overcome difficulties and strengthen their capacity to meet patient needs, although descriptions of supervision models are limited.^43^^–^^45^ A recent systematic review of LMIC-based trials of task-shared psychosocial treatments for common mental disorders found that just over half of the studies in the sample described the format, method, frequency, or cadre of supervisor used.^10^ Of those that did report these details, all conducted supervision in person; group-based supervision was more common than individual supervision; most used individual cases to guide supervision; most used expert specialists as supervisors; and most conducted supervision weekly.^10^

Few in-depth descriptions of models of supervision exist for task-shared mental health care in low-resource settings.

One well-documented approach to task-shared mental health supervision—focused specifically on psychosocial treatments—is the apprenticeship model: a collection of training and layered supervision methods originating with researchers at Johns Hopkins University, named after the model used by many crafts and trades.^46^ It is distinguished by its inclusion of 3 types of individuals: counselors, supervisors, and trainers.^47^ Counselors may be any type of mental health service provider, including community members trained to deliver a psychosocial intervention, while supervisors are counselors with the expertise or skills necessary to support other counselors. Trainers are experts from outside the service delivery context. The apprenticeship model has 5 steps: (1) selection of counselors and supervisors, (2) training, (3) practice groups, (4) supervised expansion of skills, and (5) mutual problem solving. It is relatively time and resource intensive: new counselors may shadow and observe supervisors for several weeks or months, after which supervisors perform direct clinical observation of counselor service delivery, incorporating periods of reflection and debriefing. Trainers have tended to be specialists or academics from high-resource settings.^48^ Nonetheless, investigators have successfully applied the apprenticeship model across numerous settings, including the Democratic Republic of the Congo, Haiti, Iraq, Thailand, and Zambia.^48^^–^^53^

In summary, supervision of frontline and mental health care workers falls along a spectrum from traditional supervision to supportive supervision and to mentorship. Supervisory models may vary by dosage, mode or level, tools used, and the amount of supervisor training. Limited evidence of the comparative effectiveness of these models exists. Even less evidence exists that is specific to the supervision of task-shared mental health services in low-resource settings.

## EXPERIENCES FROM THE FIELD

In an effort to further describe and learn from models for supervision that have been developed for task-shared mental health services in low-resource settings, we interviewed key informants, including researchers, program managers, and clinicians. We identified potential informants through the literature and through our professional networks, based on their experience designing, supporting, supervising, or delivering task-shared services. We used a snowball recruitment method to increase variation in position type, funder, project type, and supervisory model. We contacted potential informants by email. The first author conducted all interviews over the phone, over Skype, or in person, using a semistructured interview guide with open-ended questions. Interviews lasted up to 1 hour and covered informants' experiences with task-shared mental health interventions, the models for supervision that they developed or used, and their thoughts about how to structure future models for supervision. We digitally audio-recorded and transcribed all interviews. The first author used line by line coding to identify themes according to an inductive approach of constant comparison and content analysis.^54^ The Human Subjects Division of the University of Washington determined that this study qualified for exemption status under 45 CFR 46.101 (b).

We sought to describe and learn from models for supervision that have been developed for task-shared mental health services in low-resource settings.

Informants identified a clear need for evidence-based models for supervision that were structured, feasible, and sustainable.

We contacted 21 potential informants; 5 refused or did not respond. We interviewed 16 informants between October 2015 and January 2017. Most were researchers, and most worked in sub-Saharan Africa. Over half were from an LMIC. Informants described models of supervision from a range of different research projects or service delivery programs. Most projects and programs were based in sub-Saharan Africa, and most were funded as short-term studies by research institutes or bilateral donors. Although most offered psychosocial interventions, several incorporated pharmacological treatment. Almost all were implemented as clinical trials, led by public mental health experts based in high-resource settings, with limited funding or capacity for long-term sustainment of service delivery.

Informant experiences reflected 5 broad themes: movement from research to scale-up; building capacity for supervision by specialists; social hierarchies and supportive supervision; technological opportunities; and allowing for context, fluidity, and heterogeneity. We describe each of these below.

### Movement From Research to Scale-Up

Most of the models for supervision were designed by researchers to meet funder requirements and promote the fidelity of trial interventions. These models were often not formally structured, manualized, or designed to be sustainable or implemented at scale. Almost all informants discussed using specialists from research institutions in high-income countries, or high-resource settings, as primary or secondary supervisors, supporting service delivery through occasional calls or on-site visits. This approach has clear implications for the long-term feasibility of the programs and may limit the external validity of trial results.

*When it comes to mentoring and supervision of [counselors] over the long term, things fall apart to a certain degree because either the pilot studies that have been done they're supervised by the [principal investigator] or they are temporary. [Male Academic]*

However, some programs were moving to more scalable and sustainable models of supervision, driven in part by a shift from acceptability, feasibility, and effectiveness trials, towards implementation and scale-up research. Informants identified a clear need for the dissemination of evidence-based models for supervision that were structured, feasible, and sustainable. Commonly cited tools for this purpose included manuals, competency measures, fidelity checklists, and decision trees. Several informants described using manuals to describe and standardize supervision across supervisors and project sites and using fidelity checklists to monitor generalist practice.

### Building Capacity for Supervision by Specialists

Mental health specialists are not generally trained or paid to supervise generalists. They do not have the time or skills necessary to be supportive and offer mentorship. This limitation is especially true in low-resource settings, where specialist time is a precious resource. Specialists are trained as clinicians, not managers, yet task sharing may require them to facilitate and guide the work of dozens of generalists. Informants suggested that specialists need additional training in supervision and personnel management to manage teams of task-sharing mental health workers.

*What's very clear is, in fact, we can't just expect… people who've been trained in clinical psychology to be able to do this. It needs to be built into the core competencies for the preservice training and then they, themselves, need supervision to be able to provide supervision. It's actually caused a bit of a problem for us because the counselors are not—as far as I'm concerned—are not receiving adequate supervision because they come to the office once a week where [Program Manager] gives them supervision but it's not working very well. [Female Academic]*

Specialists need additional training in supervision and personnel management to manage teams of task-sharing mental health workers.

Although some specialists may be willing and eager to be trained to support the expansion of mental health services via task sharing, informants suggested that many are not. The specialists are often already overwhelmed with their service delivery duties, especially given the profound scarcity of human resources, and are therefore unable to add the responsibilities of generalist supervision. Some informants also reported that specialists may feel protective of their skillset and of the services they provide, acquired after years of training, and resist the notion that generalists can task share these services safely and effectively.

*One of the issues that's come up a lot in other interviews has been of special[ist] staff who are trained as service providers in psychology or psychiatry who are not comfortable diversifying their role to include supervision of task-shared health workers. [Male Academic]*

### Social Hierarchies and Supportive Supervision

Informants frequently discussed the fact that task sharing unavoidably creates or reinforces a power imbalance across the specialist and generalist hierarchies. Specialists in low-resource settings, especially psychiatrists, tend to be of high socioeconomic status and male. Generalists, such as nurses and community health workers, however, tend to be female and may be from lower socioeconomic status.

*Not only that, but you have psychologists who are trained in urban capitals, upper middle class, they don't want to work with community health workers, they speak a different language. [Male Academic]*

Informants indicated that such imbalances make it difficult for generalists to speak up during individual or group-based interactions with supervisors, making such interactions unproductive. Several informants reported the successful use of peer supervision as an alternative model that circumvented imbalances in power.

Several informants reported the successful use of peer supervision as an alternative model.

*I think the most important strength was that it encouraged the peers to really reflect and to be more alert and to contribute because sometimes what also happens is what we had noticed… is that the peers often feel intimidated. You know, they feel that when the supervisor and the expert supervisor is present in the group it's almost as though they don't feel very comfortable voicing their opinions because I guess they recognize that they're trained just specifically in one thing and they don't feel confident enough… [Female Program Manager]*

### Technological Opportunities

Several projects were implemented over large areas, often with poor roads or difficult terrain. Several informants had assumed that specialists or program managers would be able to visit generalists for on-site supervision at least once per month, only to find that such travel was not feasible. Consequently, some projects resorted to supervision at a distance, via telephone, text messages, or the Internet. Several projects found WhatsApp groups to be an effective, affordable tool for peer-to-peer, group-based supervision and discussion.

*We had a WhatsApp network with all the facilitators … They might get a reply from another colleague or other peer, but… the messages were supervised or overseen, perhaps, is a better word, by a supervisor that whenever that person felt they needed to intervene, then it will come into the network with a message for everybody. [Male Academic]*

Some projects resorted to supervision at a distance, via telephone, text messages, or the Internet.

One project used tablets to monitor implementation and service delivery and to send advice and feedback to generalists. Many successfully used video or audio recording to supervise patient encounters or therapies, sharing examples during group supervision for peer-based review and critique. Although such recording was often necessary to promote the fidelity of interventions delivered as part of research-based trials, informants suggested that it could be scaled as part of routine service delivery and supervision.

*Now that possibility would be… for the counselors to record their sessions and for those supervisors—if they're not able to go to the sites because of distances and things like that—to record the sessions and then try to do some supervision over the phone, having… listened to the recordings. [Female Academic]*

### Allowing for Context, Fluidity, and Heterogeneity

All informants emphasized that models for supervision must be adapted to the specific resources and needs of a setting. What works in one district may not work in another, and the model for supervision applied to one package of services may not translate to another package.

*I think it's context specific because there are characteristics of the context that have to be taken into consideration. I mean Ministry health infrastructure, existing staffing, rural or urban, how it's organized geographically, what's the condition of the community health workers, what's the quality of the health care system itself. [Male Academic]*

Models for supervision must be adapted to the specific resources and needs of a setting.

Moreover, informants suggested that supervisory structures have changed and will continue to change over time to accommodate the development of services and the shifting needs of caregivers.

*It's something that evolved over time. We tried quite a lot of things. We tried getting doctors to drive to the clinic and on a weekly basis work there for referrals and that just didn't work. We tried to get cases—other, severe cases—to go immediately to the tertiary facility and then that wasn't very reliable. We then realized that the best thing we could do is empower the lay health workers as much as possible. [Male Academic]*

In summary, we identified 5 broad themes. Our informants suggested that task-sharing programs are shifting from effectiveness research to scale-up; few specialists in low-resource settings have the time or skills necessary to supervise generalists; task sharing may create a power imbalance between specialists and generalists; technological solutions may improve the practicality and reduce the cost of supervision; and finally, models for supervision should be adapted to each context and will need to change over time.

## DISCUSSION

Our exploration of supervisory models for task-shared mental health care suggests that a shift is underway as programs move from effectiveness research to implementation and scale-up. Initially, investigators designed models for supervision to meet the needs of clinical trials. These benefited from the extra financial and human resources available for research, limiting their external validity, and were rarely standardized. None have been evaluated independently. As we implement programs at scale, however, it will be critical to incorporate models for supervision that are carefully planned and evidence based. We can use standardized manuals, routine fidelity checklists, and other evaluative components to promote effective and sustainable supervision of task-shared mental health services.

Task sharing requires adequate approaches to and resources for training, supervision, and emotional support.

The themes documented above align with previous studies emphasizing that task sharing requires adequate approaches to and resources for training, supervision, and emotional support.^44^ Training generalist staff to provide mental health care without considering their workloads or providing ongoing supervision can lead to inappropriate treatment.^55^ Without support, task sharing can become task dumping as generalists are overloaded with tasks they cannot perform.^56^ Where available, specialist mental health workers can be used to supervise mental health care in the primary care and community settings, although their supervisory roles must be clearly delineated and compensated.^57^^,^^58^ Sufficient numbers of specialist mental health human resources are required to provide effective and sustained supervision and support to generalists.^59^^,^^60^ Specialists should also be adequately prepared to supervise generalists through appropriate preservice training in supervision and mentorship—this aspect will require a reform in mental health specialist core competences and the engagement of specialist communities and other relevant stakeholders.^61^ In high-income countries, it is increasingly common for psychological training programs to offer courses in supervision, teaching specialist trainees the skills necessary to provide clear feedback and structured assessment of clinical practice.^62^ The American Psychological Association now considers clinical supervision to be a core competency of the health service psychologist, and it publishes guidelines for clinical supervision.^63^ In low-resource settings, public mental health services may also need to delineate generalist supervision as part of the specialist job description and establish achievable targets for the number of generalists supervised by each specialist. This approach would help specialists to have protected time for supervision and motivate them to meet their targets.

Use of established models, tools, and technology may improve the scalability and rigor of supervision, if it is adapted to context and used appropriately. The apprenticeship model offers a tried and tested method for training, mentoring, and supervising task-sharing generalists in low-resource settings, and it may improve clinical competency and provider confidence^64^—although the reliance on expert trainers may be a significant barrier to scale-up.^65^ To ensure feasibility at scale, locally available competent trainers are required. To date, trainers of programs using the apprenticeship model have often been specialists from universities in high-income countries.^48^ In the context of limited specialists in LMICs, peer-based supervision using standardized measures of task-shared provider competence may be a highly acceptable and valid complement to expert supervision, and it would help mitigate the bottleneck of limited specialists in these contexts.^66^^,^^67^ WhatsApp groups, telephone, Skype, and other technologies provide valuable spaces for individual-, peer-, and group-based supervision at a distance and on demand, improving feasibility in areas where regular in-person meetings are challenging and encouraging ongoing peer-to-peer learning.^68^ A qualitative study of supervision of community health workers using WhatsApp groups in Kenya suggested that the groups spur healthy competition and team building.^69^ However, implementers may need to be cautious with this approach and consider how best to monitor and engage in groups to ensure discussions are not misleading, distracting, or harmful. Implementers of a task-shared collaborative care model for psychotherapy in rural Nepal have shown that regular telephone calls can provide a valuable supplement to less frequent in-person supervision by a specialist.^70^^,^^71^

Adapted to context and used appropriately, established models, tools, and technology may improve the scalability and rigor of supervision.

Moving forward, it will be essential for task-shared mental health services to incorporate models of supervision that are feasible at scale and adapted to local contexts and resource levels. The Table outlines possible models for supervision according to different resource levels, expanding on a hierarchy proposed in the WHO Mental Health Gap Action Programme Operations Manual.^72^ Although we advocate for increased global investment in mental health, current approaches should be tailored to be scalable and sustainable given weak and under-resourced health systems. It will not be feasible for large programs to rely on academic researchers or experts from high-resource settings for ongoing supervision or training. Rather, programs should maximize use of in-country staff. Although the most effective models of supervision may also be the most time and resource intensive, options are available for programs with few resources. Supervision can be layered, such that experienced peers provide the bulk of supervision and scarce specialists are reserved for special cases or infrequent supervisory sessions. In many settings, it will be not be practical for supervision to occur in person. In such settings, programs could consider audio- or video-recording trainee service delivery to check fidelity, telephone or Skype calls for individual supervision, and WhatsApp or other group texts for group or peer supervision. Finally, manualized and routine supervision—supported by tools like fidelity checklists to monitor and assess provider competence—is likely to yield superior outcomes in scarce resource contexts, compared to ad hoc drop-in supervision.

**TABLE.** Models for Supervision of Task-Shared Mental Health Programs by Resource Level

Resource Level	Method	Supervisors	Level	Mode	Tools
Higher	Apprenticeship, site visit	Experts	Individual	In vivo	✓ Manualized supervision ✓ Competency measure ✓ Recording
Medium	Site visit, case recording	Experts, senior peers	Individual, group	In vivo, Phone/Skype, WhatsApp	✓ Manualized supervision ✓ Competency measure ✓ Recording
Lower	Case recording, case discussion	Senior peers	Group, peer	Phone/Skype, WhatsApp	✓ Manualized supervision ✓ Competency measure ✓ Recording

## CONCLUSION

Supervision is an understudied but critical component of task-shared mental health programs in low-resource settings. As interventions move from development to implementation and scale-up, models for supervision that are feasible for dissemination are increasingly being developed. In the absence of adequate numbers of specialists to provide supervision, technological solutions like audio recording and WhatsApp groups supported by supervisor guides and fidelity checklists can help promote better quality supervision as well as contact with supervisees. Further research is necessary to evaluate models for supervision across different programs and contexts.

Supervision is understudied, but critical to task-shared mental health programs in low-resource settings.

## Notes

**Peer Reviewed**

**Funding:** CGK was supported by grant number F31MH112397 from the National Institute of Mental Health.
